# Motivation to participate and experiences of the informed consent process for randomized clinical trials in emergency obstetric care in Uganda

**DOI:** 10.1186/s12910-021-00672-w

**Published:** 2021-07-28

**Authors:** Dan Kabonge Kaye

**Affiliations:** 1 College of Health Sciences, Department of Obstetrics and Gynecology, Makerere University, P.O. Box 7072, Kampala, Uganda; 2 Johns Hopkins University, Berman Institute of Bioethics, Deering Hall, 1809 Ashland Avenue, Baltimore, MD 21205 USA

**Keywords:** Randomized clinical trials, Emergency obstetric care, Perceptions and experiences of informed consent, Motivation for research participation

## Abstract

**Background:**

Informed consent, whose goal is to assure that participants enter research voluntarily after disclosure of potential risks and benefits, may be impossible or impractical in emergency research. In low resource settings, there is limited information on the experiences of the informed consent process for randomized clinical trials in the emergency care context. The objective of this study was to explore the experiences of the informed consent process and factors that motivated participation in two obstetrics and newborn care randomized clinical trials (RCTs).

**Methods:**

This was a qualitative study conducted among former participants of RCTs in the emergency obstetric care context, conducted at Kawempe National Referral Hospital, Uganda. It employed 30 in-depth interviews conducted from June 1, 2019 to August 30, 2019. Issues explored included attitudes about research, the purpose of the research in which they participated, motivations to take part in the study, factors that influenced enrolment decisions, and experiences of the informed consent process.

**Results:**

Respondents felt that research was necessary to investigate the cause, prevention or complications of illness. The decisions to participate were influenced by hope for material or therapeutic benefit, trust in the healthcare system and influence of friends and family members. Many were satisfied with the informed consent process, though they did not understand some aspects of the research.

**Conclusion:**

Respondents valued participation in RCTs in emergency obstetric and newborn care. Hope for benefit, altruism, desire to further scientific knowledge and trust in the investigators featured prominently in the motivation to participate. Both intrinsic and extrinsic factors were motivators for RCT participation.

## Background

Informed consent in clinical research refers to a freely-given decision or agreement for research participation following disclosure of relevant information (related to the value of participation, the procedures to be performed, the potential risks and benefits of participation and the alternatives to participation, among others). The prerequisites for a valid informed consent encompass disclosure and comprehension of information, capacity for decision-making, and voluntariness. Pregnancy and perinatal emergency care contexts pose numerous practical and ethical challenges related to disclosure, comprehension and decisional capacity and for voluntary authorization to participate) [1–7]. These challenges include inability to communicate to (and get consent from) very sick, anxious, unconscious or sedated patients, depending on how severe the patient’s condition and what medication they are taking (or have already received) at the time of recruitment. Critically ill patients frequently undergo emergency treatment that affects their cognition (and therefore capacity to comprehend disclosed information about clinical trial participation). Moreover, some conditions in emergency obstetric and newborn care (such as eclampsia, abruptio placenta, postpartum hemorrhage, obstructed labor, birth asphyxia and neonatal sepsis) not only exist mainly as emergencies which constitute the major causes of morbidity and mortality, but also make patients incapacitated to such an extent that cognition is impaired. The emergency care context also adds a stressful component to both patients and caregivers.

Several factors have been suggested as potential influences or motivation for the decision to participate in randomized clinical trials (RCTs) in emergency care contexts. These include altruism, hope for a cure (therapeutic optimism), trust in the health care system in general and investigators in particular, and having pre-formed decisions prior to invitation to participate [8–14]. A systematic review of participation in a clinical trial by pregnant women [10] found that aspirational, collateral and direct benefits, third party influence, and absence of inconvenience were factors that facilitated decision`s to participate. In this study [10], barriers to participation included inconveniences, (perceived) potential risks, and lack of trust in the research enterprise. For instance, a study that evaluated enrolment decisions of parents of former pediatric clinical trial participants found that parents went through the process of informed consent when they had already made up their minds to enroll in research [8]. This suggests that disclosure of clinical trial information may have had little influence on the decisions to participate [9].

Altruistic reasons, therapeutic optimism and a desire to get information about their children’s health are important factors that influence motivation and decisions by mothers (to have their newborns participate in research) [11]. For instance, in a newborn cohort study that investigated the theoretical acceptability of mothers to involve their newborns in clinical research [11], altruistic reasons were the main driver given for the mothers’ consent to research participation. The possibility that the mother would consent was higher if the child was healthy, if the clinical research was intended to solve a child’s specific problem, and if the perceived study risks were low [11]. Also, the most important reasons for parents’ acceptance of their children’s participation in clinical investigations, in addition to altruism, was a need to learn more about their children’s illness [12–14].

What motivates individuals to participate in RCTs in emergency obstetric and newborn care is not well documented. Expectancy theories of motivation [15] focus on the two major aspects of motivation, *direction* (which alternatives) and *intensity* (how much effort to implement the alternative). These theories posit that the attractiveness of an alternative is determined by “expectations” of what is likely to happen if it were chosen, such that the more individuals believe that the alternative chosen leads to positively valued outcomes, the greater its attractiveness. From the theories, motivation is the process that initiates, guides, and maintains goal-oriented behaviors [15]. Motivation for this study was defined as the compelling reason(s) an individual to enroll in RCTs in emergency obstetric care. The theoretical framework for this study was the socio-ecological framework, where individual’s motivation for enactment of a behavior is influenced by intrinsic factors or extrinsic factors. Intrinsic factors are factors within the individual (such as personal need for healthcare, knowledge, attitudes, perceptions, beliefs and misconceptions). Extrinsic factors include interpersonal interactions (such as motivation to help others), and immediate context (family community or society). The objective of this study was to explore factors that motivate participation in RCTs in emergency obstetric and newborn care.

There is a need to evaluate and delineate factors that contribute to participants’ motivation to enroll and continue participation in research in the emergency obstetric care context. Whereas some factors are documented for some non-clinical trial studies, or for some RCTs in specific populations such as children, patients with cancers and patients with chronic illnesses, there is little published on clinical trials in emergency obstetric care, and more so in resource-limited settings such as Uganda. This study sets out to explore factors that would motivate individuals to participate in RCTs in the emergency obstetric care context.

## Methods

### Study setting and participants

#### Study setting

This study was conducted in the postnatal clinic of Kawempe National Referral Hospital. This hospital is a 450-bed specialized obstetrics and gynecology referral hospital located in Kampala, Uganda. Data collection occurred from June 1 to August 30, 2019 and involved in-depth interviews with 30 former research participants of two randomized clinical trials in emergency obstetric care. Participants were contacted during their postnatal visit (at least 6 weeks after suffering severe obstetric complications during pregnancy or childbirth). The inclusion criteria were women aged 16 years or older, having participated in an RCT during emergency obstetric care (during pregnancy or childbirth), and having been recruited in the clinical trial when the participants had a severe obstetric complication.

#### Study participants

Participants were recruited from 2 RCTs: a) 10 were recruited from the Springfusor study (NTC03549767), a parallel assignment randomized clinical trial of acceptability of a device to administer loading and maintenance doses of Magnesium Sulphate injection in patients admitted with severe preeclampsia or eclampsia, between June 2018 and August 2019. Here 241 women recruited at admission were randomized to receive either Magnesium sulphate (standard administration) or the intervention (using an infusion pump device). The endpoints were acceptability, safety and efficacy of the intervention. b) 20 were recruited from the Phone Call and Message Text study (PACTR201907640298298), an RCT used to assess the effect of telephone calls and text messages on postnatal clinic attendance among 488 post-caesarean section mothers at Kawempe regional referral hospital, Uganda, where 488 women were recruited before performing emergency caesarean section for obstructed labor. These were randomized to two arms: to receive telephone call and phone text messages reminders (to attend postnatal clinic at 7 days and six weeks postpartum), or to receive the standard of care (verbal information to come for postnatal review at 6 days and six weeks postpartum at the time of discharge. The study endpoints were acceptability of the phone text messages and proportion of mothers who returned to the health facility for postnatal review as recommended. Twenty women were interviewed from this study.

### Data collection procedures

To verify eligibility, the investigator assessed the patients’ medical records to confirm that they had suffered a severe obstetric complication (severe preeclampsia or eclampsia or severe obstructed labor) and participated in either the Springfusor study or the Phone text messages RCT. Potential participants provided written signed informed consent after being provided with detailed information about the study, which was reviewed verbally with each individual. Participants were given assurance that they were free to decline, that even if they declined, their decision would not affect the care that they were entitled to from the postnatal clinic, and that information provided would be kept confidentially. While all respondents understood both languages, the interviews were conducted in English and Luganda and the interviews were audio recorded and field notes were taken.

Issues explored included attitudes about research, the purpose of the research in which they participated, motivations to take part in the study, perception of the research procedures, especially randomization. We also explored factors that influenced enrolment decisions, experiences of the informed consent process, especially perceptions of potential risks, benefits and alternatives to participation.

### Data analysis

The NVivo 10 a software package was used to manage the data. Data analysis involved evaluation of audio recordings and field notes to transcribe the data. The transcripts were analyzed by thematic analysis described by Mays and Pope [16, 17]. Data analysis employed the constant comparative method to analyze for codes or meaning units (recurrent patterns statements, words or phrases with similar meaning or interpretation) across data set [18–21]. The codes were aggregated into themes (groups of word patterns or phrases with similar meaning) to provide a description of the experience of the phenomenon (motivation to participate in randomized clinical trials during emergency obstetric care). Representative quotes from participants derived from the individual transcripts were included to illustrate the source of interpretations of information.

### Ethical considerations

Permission to conduct the study was obtained from the Department of Obstetrics and Gynecology, Makerere University. Ethical review for the study was obtained from Makerere University School of Medicine Research and Ethics Committee (SOMREC), Mulago Hospital Research and Ethics Committee and from Uganda National Council of Science and Technology (SS 4952). The studies in which the participants had participated earlier had received approval from relevant ethics committees as applicable, and were registered as RCTs. All participants gave written informed consent to participate. This consent was obtained at the time of the postnatal visit. Any information shared about unpleasant experiences from previous obstetric complications was discussed in an empathetic manner. All participants were aged 16 years and above. Participants who seemed emotionally affected by recall of unpleasant experiences during pregnancy or childbirth were offered counselling. Participants were given assurance that they were free participate and that even if they declined, their decision would not affect the due care that they were entitled to.

## Results

There is a need to evaluate how subjects understand and experience their participation in emergency care research, particularly clinical trials, in order to delineate factors that contribute to participants’ motivation to enroll and continue participation in research in this context. The study shows that research participants’ motivation to participate in emergency clinical trials, decisions to volunteer for research studies and reasons for their motivation to continue participation are often multi-faceted and may be impacted by several factors. These include their medical condition, consideration of their situation and a multitude of internal and external factors.

### Personal and intrinsic factors in relation to motivation to participate in RCTs

Many participants believed that their participation in research was a good decision taken, giving several reasons why they found enrollment in research acceptable. These include the necessity to learn more about illness and the need to assess new ways of delivering healthcare, or for altruistic reasons, where medicines tested may not benefit the participant, but may be of benefit to other patients in future as exemplified by several respondents:

Respondent 1 (Preeclampsia study): “Sometimes, the cause of the illness is not understood… Even the doctors do not have answers… There is a lot that is not known…(Pause) I think this can help find some of those answers…Can help get solutions”.

Respondent 2 (Phone text message study): I think it is necessary…Where you know that getting information (to treat the illnesses) requires pregnant women to be involved...to contribute to the solution…That is something right to do.

Respondent 3 (Preeclampsia study): “I think it may be necessary to know what doctors need to know… or test new ways of giving treatment like in our study…There is a lot we do not know. ..It would be good to get answers to these questions… I would be very willing to take part…It is also good to help other mothers, if the information helps other mothers, it is very good on my part...I would know that I helped somebody.”

Respondent 9 (Phone text messages study): It is very important…(It) may help you know about what made you sick… They may find evidence on how your baby may be affected by illness… I think they are necessary.”

Respondent 10 (Preeclampsia study):” I think it is important… It is good to check if there are new medicines…may even assess alternative medicines (Pause) It can help identify medicines for the future, even if I may not benefit personally.”

Several reasons were given as the motivation for research participation. These included the need to get some form of benefit, such as better attention and care and free medication:

Respondent 6 (Preeclampsia study): “I am grateful that they, as promised, provided some drugs that I could not afford…This (promise) was a strong encouragement for me.”

Respondent 14 (Phone text message study): “The expectation that someone would be checking on me using a phone call or text message to remind me about my healthcare was very important… it showed that the nurses were concerned about my wellbeing… By showing they care, they encouraged me to take part.”

Both therapeutic optimism and hope for material benefits are motivators for participation in research. The influence of perceived material and health-related benefits, suggest that investigators should pay attention to planning for possible material benefits for research participants as incentives to join the study, as exemplified by two respondents:

Respondent 15 (Preeclampsia study): “The care given to me was an encouragement (narrated her experience) My (blood) pressure was very high (due to severe preeclampsia). There were many investigations they did before they told me my situation was complicated and urgent. I asked some questions and they answered me…I kept wondering why me? Why now? What did I do wrong? It had not happened before…But they were supportive…then I thought, taking part may help find answers to why the illness occurs…may find evidence why diseases don’t improve …I was eager to take part…it was good somebody was to meet the treatment costs…that is all I have to say.”

Yet for others, the motivation was for altruistic reasons, related to the desire to contribute to the generation of evidence for advancing scientific knowledge, as exemplified by two participants*:*

Respondent 18: (Preeclampsia study): “To me I think it is important…the only way to understand difficult diseases like high blood pressure…what causes them? Why do they happen? What do you do to prevent them? I think these are relevant questions. If research provides answers, then it is right. That is why I agreed to take part…if the research generates evidence on what medication to use, then it is okay. (Pause) Actually, I would not miss that opportunity if I got another chance.”

Respondent 1 (Phone text messages study): “If the disease is very severe, one has few options… if the participation is offered, why refuse? … They were eager to help me…this may be a new approach to providing care…It may viable alternative to provide help for oneself and for others.”

### Experiences of the informed consent process in relation to motivation for RCT participation

The participants described dissimilar experiences regarding how they ended up as research participants in the preeclampsia study, and how these experiences may constitute barriers or motivation to research participation. Some of the participants reported some deviation in study procedure from the information that had been disclosed to them. Whereas some signed an informed consent first (before any procedures were done), others reported that they signed a form after some study procedures had been performed, possibly as part of emergency resuscitation before recruitment into the study. For some participants it was difficult to remember where the routine care ended and aspects of research procedures began. These views are exemplified by these respondents:

Respondent 2 (Preeclampsia study): “ I was given information about the study soon after admission…I don’t seem to remember much of what they told me… I remember they wanted me to be part of a study…I was not feeling well at the time… the abdominal pain was very severe…they gave me some medication to reduce pain, but the dizziness remained…I could not see clearly…they assured me all women with my condition are given the same treatment as what was to be given to me… that is all I recall.”

Respondent 3 (Preeclampsia study): “I can remember being given some information... I also remember them asking me to join… I had already received some treatment…I do not remember the details, but I remember that I agreed, though I was not very sure what was to be involved… I also remember signing a form (using a thumb print)..I only vividly remember the pain from the injections...Yes, I also remember we (patients) all got the same treatment that day...Oh the injections really hurt…but I improved soon after the treatment.”

The study findings also suggest some potential barriers to research participation in clinical trials in the emergency care context. These include failure to communicate the clinical trial information to individuals who may not be in position to understand:

Respondent 3 (Preeclampsia study): “they had to wait until I was somehow better, when could understand... they read the information to me… but I do not remember much…They left some information on a leaflet on my bed…I took my time… I need to read and understand for myself”.

Many participants experienced difficulty in understanding the informed consent process. Deferred consent using verbal consent prior to randomization with written consent completed after the randomization process was used for some patients. On whether the respondents would agree to participate without going through the informed consent process, most participants expressed no major objection. The reasons for this was that investigators did not appear discriminatory in the choice of who was to participate among those with similar illness (phone text messages study). Also, they did not think that participation involved serious risk or inconvenience (considering that the medication given in the preeclampsia study is the same as that given to all patients with the condition).

Respondent 24 (Phone text message study):“How they went about it did not bother me…After all, they assured me that I was free to decline at any moment that I chose...What they were asking me to do did not make much sense to me…that this was not common practice to call patients to remind them of hospital visits…they made it clear joining was voluntary…so I said why not…if they were going to call every day or many times every day, it would be a different issue… I wouldn’t accept that.”

### Investigator behaviors and influence on the motivation for RCT participation

For most participants, the experience of the informed consent process was positive, as they reported that they were approached at a time when they had already received attention and care (emergency anti-hypertensive therapy for the preeclampsia study). Therefore, investigators’ prior participation in the process of care giving was one of the motivators for RCT participation, as it seemed to nurture trust. This influence by investigators and clinical care givers, who are part of the emergency care contexts, is exemplified by three respondents:

Respondent 2 (Phone text message study): “They were kind…took time to explain what they wanted from us…asked if we understood… did not put you on pressure to accept.

Respondent 3 (Preeclampsia study): “They seemed genuine… they promised compensation if something went wrong…they indicated that we are free to choose to take part, but the group would be one way or the other according to your chance…I think this got me even more interested in taking part.”

Respondent 22 (Preeclampsia study): “They treated us really well…they really gave us attention…showed they were caring...They were kind… provided all the medication…they did not seem to be in a hurry to get you sign their forms… they asked if you understood what they wanted you to do… I did not understand much when they came to me…I did not hesitate when they asked me (to join).”

### Extrinsic factors in relation to motivation for RCT participation

In this perspective, individuals found that the decision for RCT participation was made not by the individuals but by family members, who included spouses. This shows that to some participants, the views of their spouses and care givers- who for many are not usually family members-contributed to the RCT participation decision, and this was found both important and acceptable. The decision-making process required (in some cases) advice contributed by the spouses, caregivers or friends. This is exemplified by two respondents:

Respondent 15 (Preeclampsia study): “For me I was unwell… it is my husband who signed the form first accepting that I should be operated…he also signed on the form that I take part (in the study). I think this was important…he needed to know all that was happening to me… his agreement was important for me…even the doctors took their time to explain…I had many questions, but they tried their best to give answers…the final decision was mine, but all needed to agree to what was happening”.

Respondent 6 (Preeclampsia study): “Personally, when I was told about the research the

very first time, I felt uncomfortable...I had to ask some friends… they told me it was okay to consult others… to get their opinion. Now, my thinking is that they supported me in taking the decision…my body was tortured (was in the standard of care treatment arm), but at least I had consulted before accepting to take part…was even reassured that this is the treatment all patients have to get…such advice is important….it

Many participants had difficulty in making the decision to participate, and some felt that it is not the information presented to them at the time of recruitment that contributed most to their participation decision. Rather, prior conviction that they should not miss a chance to participate (as long as an opportunity was available) or advice from relatives or friends were the motivating factors, as exemplified by one respondent:

Respondent 2 (Preeclampsia study): “It was not a simple decision to make… I had to consult my spouse…I also consulted some friends first… Much as you have the final say, advice is important… (Pause) For instance, you need your husband to give his view on the decision... Advice from friends was crucial... (Probed which persons to consult and why)…these are people I trusted…who were concerned about my health…I needed their advice…They needed to know what was going on”...This is the right thing to do...before you make the final decision.”

Also, many participants reported that the information got from other individuals had significant influence on their decision to participate, and this information was received prior to the invitation to participate and disclosure of the clinical trial information. This highlighted the importance of communal decision-making and solidarity when it comes to making decisions to participate in emergency obstetric care research.

Respondent 3 (Preeclampsia study): “I was in a seated on the bed and feeling dizzy. Then I started vomiting. Then one mother said ‘you may be having high blood pressure…’. She called the nurses. They took my blood pressure and gave me some injection, as it was high…then the mother who called the nurses said that she was in a study where they treat mothers with a problem like mine…I asked her how and why she joined…then she said that the injections were painful, but in the study, you get a chance to get medicine by a different method…the advice was very useful…it made it easier for me to accept.”

Thus, some respondents valued the support in making the decision to participate, reporting that much as it was them that made the final decision, they widely consulted family members, other mothers or friends, before making the decision:

Respondent 24 (Phone text messages study): “I was afraid when approached…Now like myself, I personally have never been told about research before… did not know why they singled me out…I value my privacy…But when I asked other mothers, they said why not? What was I going to lose…I think their advice was important…otherwise I was going to reject it (participation).”

helps to decide.”

## Discussion

The study findings show that both extrinsic and intrinsic factors motivate participation in clinical trials in the emergency obstetric care context. The expectation of personal benefit (such as opportunity to access treatment or motivation) and the hope for others’ benefit (such as generating new knowledge about illness) are in agreement with the expectancy theories of motivation [15]. From the expectancy theory of motivation, individuals are motivated by the personal interest or for pure enjoyment of the activity or task [15]. Participants who were motivated by a desire to help others, not necessarily for own benefit, demonstrated intrinsic motivation (that is, the act of doing something without any obvious external rewards). Intrinsic motivation comes from within the individual, and individuals who are intrinsically motivated engage in an activity solely because of enjoyment and desire for personal satisfaction [15]. In contrast, participants who were motivated by a need to access some benefit (some form of external reward) demonstrated extrinsic motivation.

The study highlights the complexities of recruitment of participants for RCTs in emergency obstetric care contexts. Modification of the informed consent process as practiced in non-emergency clinical trials may be required. Some of the modifications include waiver of consent, deferred consent, surrogate consent, targeted consent and advanced consent [1, 2, 6, 22, 23]. Waiver of consent may be indicated where some emergency treatment interventions or procedures must be delivered immediately, and yet the emergency contexts does not allow opportunity to obtain consent prior to enrollment. This could have been the case for the preeclampsia/eclampsia study, where treatment may be required as part of emergency resuscitation or emergency treatment, and needs to be given before informed consent procedures are initiated or completed. Deferred consent involves randomization according to explicit criteria approved prior during ethical review of the protocol, at the investigator’s discretion followed by a future request for the patient’s (deferred subject con-sent) or representative’s (deferred proxy consent) informed consent [22, 23]. Some participants described some form of proxy consent, where the spouses and care givers took a major part in the decision-making or made the decision to participate. What some of the participants in the preeclampsia appeared like deferred consent, since (and which may be justified by the fact that) they were not in position to provide informed consent at the time of admission, but investigators wanted them to take part. They could have been recruited into the study and study procedures initiated before full enrollment. While the latter was perceived by some participants as against autonomous decision-making (in line with self-determination theory of motivation), advanced consent provides an opportunity to obtain informed consent from populations with risk factors for certain emergency conditions such as severe pre-eclampsia [24, 25].

The findings are in line with the self-determination theory of motivation [26], which posits that while individuals yearn to be autonomous (that is, need to be in control of their own behavior, destiny or goals), they need (and seek) connection or relatedness [26]**,** that is, they need or experience a sense of belonging and attachment to other people. From this theory, individuals let others contribute to the decision-making process, as a result of their desire for connectedness. Whether individuals are proactive and engaged or passive and alienated depends largely on the situation and the context, that is, the social conditions and the environment respectively in which the decisions are made [26]. From this study, the evidence of participants displaying extrinsic motivation include need to contribute to finding information that may help doctors address the medical illness or problem, the need to generate information which may not primarily help participants but may help others, and need to advance the existing scientific knowledge about obstetric illness, or the need to assess effectiveness of phone call and text messaging in healthcare. Extrinsic motivation, in contrast to intrinsic motivation, refers to the motivation to do something in order to attain some external goal or meet some externally imposed constraint [27].

In line with both the expectancy and self-determination theories of motivation, the study findings show that the context may influence motivation to participate. Decision making for participation was challenging to both groups of participants. However, respondents from the phone message text study had less challenge in reaching a quick decision to participate than participants from the eclampsia study. This difference may be dependent on the perceived risk versus benefit of participation, as well as the perceived impact of the decision taken. While invitation to participate in research may always carry a degree of uncertainty and general fear, this feeling may be short-lived for some studies depending on perceived risk (making it easier to make a quick decision to participate), as a manifestation of autonomy in motivation [15, 26]. However, this fear may be stronger or longer-lasting, leading to initial hesitancy that may require consultation of significant others about the decision (in relation to the need for connectedness) [26] with later acceptance of participation. Thus, in line with both the expectancy theories [15, 27] and the self-determination theories [26], motivation for emergency care RCT participation depends individual autonomy and circumstances, including prior preferences, perceived need, perceived risks and perceived benefits.

In line with the expectancy theories of motivation [15], individual are inclined to behave or act in a certain way because they are motivated to select a specific behavior over others depending on what they expect the result of that selected behavior will be. Thus, individuals are motivated to satisfy own needs (such as need for medication or material benefit or need to help others), positively value outcomes that satisfy unmet needs, negatively value outcomes that curtail satisfaction of unmet needs (such as risks associated with research participation), and allocate neutral values to outcomes that do neither. From this viewpoint, the desire for material benefit, medication or provide help to others (other than self) may motivate participation in RCTs. In contrast, RCT participation risks may be perceived as negative value outcomes, which may demotivate research participation. Where no direct risk or benefit exists (such as no direct benefit or harm from receiving phone call or message text reminders) may constitute neutral value outcomes. Where motivation refers both to the triggers of the behavior and the factors that maintain or direct goal-oriented actions, the study findings indicate that individuals’ interactions with their environment influences motivated behavior for participation in emergency care RCTs.

The findings have implications for recruitment of participants into emergency care research. From the expectancy theory [15], the motivation of a given behavior selection depends both on both the cognitive process of how an individual processes the different motivational elements and how desirable the perceived outcome is. Therefore, how and what information is disclosed, the mental processes regarding choice, and the processes that an individual undergoes to make choices matter. Adequate information about the RCT related to the purpose, procedures, potential risks and benefits (which relate to extrinsic rewards) should be disclosed. From the self-determination theory [26], disclosure enhances autonomous decision-making (enhances ability of individuals to make choices according to their goals, interests and preferences). However, extrinsic rewards should be used sparingly as they can potentially undermine intrinsic motivation when used in certain situations. Benefits pose risk of undue inducement (if they interfere with processing information about risks versus benefits), if are excessive, or may be perceived as coercive (if participants have limited choices).

As noted in the study, some participants were not in position to understand whether they were research participants until later when they recovered, yet had provided consent. There is little consensus regarding what level of understanding is needed for informed consent to be valid [28–30]. Considering that some participants were eager to participate even before invitation, it is unclear whether the participation decision was influenced by the disclosed information. Therefore, it is unclear whether decision making for research participation should depend primarily on the disclosed information rather than participants’ views and values [31, 32]. Normative principles relating to how consent *ought* to be obtained in emergency care research contexts raise important and difficult ethical questions [28–34]: For instance, how should the information be disclosed to prospective participants (some of whom may be unable to comprehend the information)? Is comprehension of the disclosed information) necessary (especially if participants can demonstrate adequate knowledge before or participants do not use this information for decision-making) ? If yes, is it possible to always achieve comprehension in emergency research? Do prospective participants use the disclosed information to make decisions about research participation? Can there be a truly voluntary decision to participate in emergency research? What is the minimum information that participants should understand before their decision to participate is taken as valid, or to guarantee an informed consent during their participation? [33, 34] All these are complex ethical issues that relate to motivation for participation in emergency research.

The reliance on investigators, spouses or caregivers for either motivation or the decision to participate shows the importance attached to trust and connected ness in motivation theories [26]. Trust relates to connection [26]. Trust is perceived as a crucial feature of care that has to be “protected and nurtured in order to improve people’s experience of medical services and their overall health” [35]. Trust is also perceived in a broader view as “ all manner of relationships, including trusting oneself, one’s body, the health service, and other significant people… all these forms coalesce around a person at times when they feel vulnerable and try to make sense of their situation by locating themselves in a network of relationships that might sense of stability”[35]. Thus, trust is called upon by individuals at a time of need or vulnerability to see how they cope, adapt to or overcome the uncertainty induced by certain situations on their life [36, 37]. Trust is a key cornerstone of effective doctor–patient relationships, and relates to the vulnerability associated illness, information asymmetries arising from the investigator-participant relationship at the time of recruitment into research, and from the perceived uncertainty and risk associated with intentions of the practitioner (or investigator) on whom the patient is dependent [38]. At the institutional level, trust in hospitals and health care systems may affect patient support for and use of services (or even research participation). [38]. The consequences of such trust may include insufficient comprehension by research participants of the inherent logic of clinical trials, the choice of trust over comprehension (prioritization of trust), and reliance on motivated reasoning for decision-making [38]. In the emergency research context, certain researcher behaviors can persuade, manipulate, or coerce potential research subjects. The study shows that several factors influence a person’s motivation to take part in a trial in the emergency obstetric care context. These include the researcher’s status as holders of positions of status, trust or influence. This may operate through referral or advice on research participation, especially in situations where potential participants have limited choices about alternatives to (obtaining care other than from) research participation [39, 40]. Investigators should consider some of these when recruiting participants, and should understand that potential participants have different expectations and motivating factors [41–43]. Failure to address these may constitute barriers to recruitment or retention of research participants.

Regarding limitations, only one data collection method and one data source (former research participants) was used. Triangulation of data sources and data capture methods can enrich and provide deep understanding of a complex phenomenon like the informed consent process, which may need assessment of investigator and participant perceptions and behaviors as well as contextual factors to corroborate the findings. Secondly, while a systematic process was used to analyze the data, analysis was conducted by the researcher alone, which limits credibility of the data interpretation. Thirdly, where motivated behavior is mentioned, this is self-reported information, and therefore subject to bias including social desirability bias. Observational prospective research could reveal more detail about the participant experiences and perceptions, as well as any deviations of actual practice from perceived practice. Lastly, respondents were restricted to former participants of RCTs, whose views and perceptions may not be representative of those not invited to participate, or who were invited but declined. However, this study employed purposeful sampling to recruit participants who shared experiences of suffering an emergency obstetric complication, being invited to participate in research in an emergency care situation, and having accepted RCT participation. The issue that was interrogated here was participants’ experiences on being invited to participate in relation to motivation to participate in RCTs in emergency care. Besides, in-depth interviews allowed capture of participants experiences, perceptions, values and meanings attached to reported practices, views and motivation (where motivation is a goal-directed behavior). Also, using a specific detailed data analytical framework and presentation of quotes supported the interpretations of the findings. Besides, qualitative studies overcome some of the limitations of quantitative research data on motivations for research participation.

## Conclusion

Both extrinsic and intrinsic factors motivate participation in clinical trials in the emergency obstetric care context. The need for treatment, hope for own benefit or curiosity and eagerness to learn about the illness indicates intrinsic motivation, and limited opportunities for healthcare represented extrinsic motivations. Participation decisions were constrained by the participants’ ability to understand disclosed information about study procedures and the interventions, but were positively influenced by altruism, hope for personal benefits of taking part, trust in the investigators and solidarity. The participants reported a positive experience of the informed consent process, and felt that the timing of the enrollment at a specific moment in the care continuum matters. Also, different approaches were acceptable to seek informed consent from participants. Respecting persons, in the context of clinical research in emergency contexts, includes recognition of and respect for the diverse cultural values. A valid informed consent in an emergency context may necessitate adapting unique and specific practices in relation to disclosure of clinical trial information, assessing complication and obtaining authorization for research accordingly.

## Data Availability

The data is available on request from the corresponding author.

## Abbreviations

RCT: Randomized Clinical trial
REC: Research and Ethics Committee
IRB: Institutional Review Board
SOMREC: School of Medicine Research and Ethics Committee
